# Acceptability of COVID-19 vaccine: a cross-sectional study in a Tunisian general hospital

**DOI:** 10.11604/pamj.2021.39.245.27199

**Published:** 2021-08-17

**Authors:** Hamdi El Kefi, Khira Kefi, Mohamed Wassim Krir, Chaker Bencheikh Brahim, Abir Baatout, Imen Bouzouita, Mouna Ben Azaiz, Chaker Bouguerra, Mohamed Taha Khoufi, Hedi Gharsallah, Hela Slema, Abdelaziz Oumaya

**Affiliations:** 1 Psychiatry Unit, Military Hospital of Instruction of Tunis, Tunis, Tunisia,; 2 Immunology Laboratory, Military Hospital of Instruction of Tunis, Tunis, Tunisia,; 3 Preventive Medicine Unit, General Directorate of Military Health, Tunis, Tunisia,; 4 Intensive Care Unit, Military Hospital of Instruction of Tunis, Tunis, Tunisia

**Keywords:** Vaccination refusal, coronavirus, hospital personnel

## Abstract

**Introduction:**

the year 2020 was marked by the COVID-19 pandemic that killed more than one million people. Several vaccines have been developed and vaccination campaigns started in December 2020. The objective of our study was to assess the acceptability of the COVID-19 vaccine by hospital staff.

**Methods:**

cross-sectional study conducted on a representative sample drawn at random from the staff of the Military General Hospital of Tunis. Data was collected between August and September 2020 by a clinical psychologist. We studied the associations between the different characteristics of our population and the decision to accept or refuse vaccination against COVID-19.

**Results:**

a total of 398 hospital staff agreed to answer our questionnaire. Our sample was composed of 9% (n=36) physicians, 0.9% (n=3) pharmacists, 41.3% (n=164) paramedics, 16.1% (n=64) cleaning staff and 32.7% (n=131) administrative staff. The rapid discovery of the vaccine was hoped by 97% (n=386). Vaccination was considered a means of collective protection by 84.7% (n=337). However, only 58% (n=231) agreed to be vaccinated by the COVID-19 vaccine. The main factors significantly associated with acceptance of the COVID-19 vaccine was previous influenza vaccination (aOR: 2.58, 95% CI 1.69-3.94; p=0.000).

**Conclusion:**

apprehension about vaccination does not appear to be sparing the future COVID-19 vaccine. Fear of vaccine side effects outweighs fear of the disease, even among hospital staff. To achieve vaccination coverage, several awareness and communication activities must be carried out.

## Introduction

On March, 11^th^, 2020, the spread of COVID-19 was first described as a pandemic by the director general of the World Health Organization (WHO). Since that date, the number of infected people has continued to increase in the world and in Tunisia. As of 26^th^, June, 2021, 179,075,604 cases have been reported in the world and 403,493 in Tunisia [1,2]. The number of deaths was 3,876,675 in the world and 14,579 in Tunisia [1,2].

At the time of writing this article, 7 vaccines against COVID-19 have already been authorized by the WHO in addition to a few under development [3,4].

Vaccination campaigns began in December 2020 to reach on June 26^th^, 2021, 2.92 billion doses administered with 10.2% of the world population fully vaccinated (2 doses) [5]. In Tunisia, 1.69 million doses were administered with full coverage of 3.9% [5].

Vaccination strategies against COVID-19 were drafted, following the example of the possible vaccination scenarios and preliminary recommendations on target populations developed by the High Authority of Health (HAS) in France [6], and the guidelines to plan for COVID-19 vaccine introduction of the WHO [7]. In all scenarios, it is recommended to vaccinate front-line health first and medico-social professionals. The objectives are to guarantee individual and collective prevention and to maintain these essential activities during epidemic periods [6-9]. It is important to understand the barriers and promoters to vaccination of healthcare workers to improve strategies and interventions to promote vaccination against COVID-19. This study aimed to assess the acceptability of the COVID-19 vaccine by hospital staff.

## Methods

**Study setting, study design and study population:** we conducted a cross-sectional study on a representative sample of the staff of the Military Hospital of Tunis (doctors, pharmacists, nurses, orderlies, cleaning staff, technicians and administrative staff). We asked the hospital staff included in the study to respond to a questionnaire that we specially developed.

**Data collection and study definitions:** data collection was carried out during the months of August and September 2020 by a clinical psychologist. Our questionnaire, in addition to socio-demographic data (sex, age, position, number of years of service), included questions on influenza vaccination status, the psychological impact of the COVID-19 pandemic, factors that could reduce the spread of this virus, acceptance or refusal of future vaccination against COVID-19 and factors justifying or influencing this choice. The questions are closed-ended with the possibility of proposing an alternative answer if no proposal is appropriate.

**Sample size and statistical analyses:** we estimated the minimum sample size at 368 individuals, for a t confidence level of 95%, a margin of error of 5% and a p risk level of 40%. From the list of all hospital staff, 400 participants were randomly selected. These participants were contacted to complete the questionnaire in the order of exit from the draw.

We calculated absolute and relative frequencies for the qualitative variables. We also calculated means, extreme values for the quantitative variables and standard deviations. We then performed univariable and multivariable logistic regression to identify socio-demographic and clinical factors associated with reluctance of the COVID-19 vaccine. Variables with a P-value ≤ 0.2 in univariable models were included in multivariable models through backward stepwise method. Variables in the final model with p<0.05, were considered statistically significant.

**Ethical considerations:** the study took place after approval from the local ethics committee of the Military Hospital of Tunis (reference number: 67/2020/CLPP/HMPIT). We obtained the free and informed consent of all the participants.

## Results

**General characteristics:** a total of 398 hospital staff agreed to answer our questionnaire. They were 55.3% (n=220) women and 44.7% (n=178) men. The average age was 40.5 years (22-60 years) with an average number of years of service of 17.04 years (1-38 years). Table 1 shows the distribution of our study population by function.

**Table 1:** population distribution by function

Function	n (%)
Doctors	36 (9)
Pharmacists	3 (0.9)
Nurses	111 (28)
Health technicians	33 (8.3)
Orderlies	20 (5)
Cleaning staff	64 (16.1)
Administrative staff	131 (32.7)

Two hundred and three participants (51%) never had influenza vaccination, 26.6% (n=118) were vaccinated occasionally, 19.3% (n=77) were vaccinated annually. Vaccination was considered a collective protective act by 84.7% (n=337) of the participants, while 15.3% (n=61) considered it an individual protective act. Only 27.4% (n=109) of the participants had direct contact with patients with COVID-19. Anxiety symptoms (anxiety, insomnia, irritability, anxious ruminations) occurred in 73.4% (n=292) of participants, 26.6% (n=106) said they were indifferent.

**COVID-19 vaccine reluctance and reasons:** three hundred eighty-six (97%) of respondents hoped for a rapid discovery of the COVID-19 vaccine. Two hundred and thirty-four (58.8%) agreed to be vaccinated for the following reasons: 92% (n/N=215/234) to protect themselves and their families, 2.6% (n/N=6/234) because they believe in vaccination, 5.4% (n/N=13/234) because they believe that vaccination is mandatory for health workers.

The reasons for reluctance to vaccination by 41.2% (n=164) participants were the fear of side effects for 57.3% (n/N=94/164), the doubts about the vaccine's efficacy for 30.5% (n/N=50/164) and the reluctance of any vaccination 12.2 % (n/N=20/164). Fear of possible side effects of the new vaccine was the main cause of refusal for all professional categories (Table 2). Factors that could make them change their minds were the certainty that the vaccine was safe for 48.2% (n/N=79/164), the significant spread of the virus for 11% (n/N=18/164), and mandatory vaccination for health personnel for 9.8% (n/N=16/164). The refusal was final for 31% (n/N=51/164). According to the hospital staff, the factors that could limit the spread of the virus and protect us are shown in Table 3.

**Table 2:** causes of refusal of COVID-19 vaccine by academic background and function

	Causes of reluctance of COVID-19 vaccine (n=398)			p
	**Fear of side effects, n (%)**	**Doubt about efficiency, n (%)**	**Against all vaccinations, n (%)**	
**Academic training**				
Medical and related	59 (59.6)	33 (33.3)	7 (7.1)	0.044
Non-medical	35 (53.8)	17 (26.2)	13 (20)	
**Function**				
Physicians and pharmacists	15 (68.2)	5 (22.7)	2 (9.1)	0.043
Paramedicals*	44 (57.1)	28 (36.4)	5 (6.5)	
Cleaning staff	9 (42.9)	5 (23.8)	7 (33.3)	
Administrators	26 (59.1)	12 (27.3)	6 (13.6)	

* Paramedicals: nurses, health technicians, orderlies

**Table 3:** factors that hospital staff believe can limit the spread of the virus

Factors	n (%)	OR (95% CI)	p
The adapted strategy to fight COVID-19	267 (67.1)	0.99 (0.62-1.59)	0.983
Divine protection	92 (23.1)	0.75 (0.49-1.15)	0.195
BCG vaccination	69 (17.3)	0.83 (0.49-1.40)	0.491
Tunisian diet	50 (12.6)	0.87 (0.48-1.59)	0.668
Genetic factors	45 (11.3)		
Tunisian climate	23 (5.8)	1.36 (0.55-3.22)	0.519
Viral mutation	21 (5.3)	0.62 (0.25-1.49)	0.285

CI: confidence interval; OR: odds ratio

**Correlates of COVID-19 vaccine reluctance:** in the univariable analysis, we found no significant influence of sex, age and years of service on whether to be vaccinated against COVID-19. However, those most willing to be vaccinated against COVID-19 were those who were already vaccinated against influenza (OR: 2.59, 95% CI 1.70-3.98; p=0.000) and administrative staff (OR: 1.37, 95% CI 1.01-1.85; p=0.03). The most reluctant to vaccinate were those with medical academic training (OR: 0.53, 95% CI 0.35-0.80; p=0.002) such as physicians and pharmacists (OR: 0.52, 95% CI 0.25-1.05; p=0.067) and paramedics (OR: 0.66, 95% CI 0.44-0.99; p=0.038). After multivariable analysis, the only factor significantly associated with COVID-19 vaccine acceptance was previous influenza vaccination (aOR: 2.58, 95% CI 1.69-3.94; p=0.000) (Table 4). Participants' feelings anxiety or indifference (OR: 1.46, 95% CI 0.93-2.29; p=0.092) and the beliefs about the factors influencing the spread of the virus had no significant influence on the acceptability of the COVID-19 vaccine (Table 3).

**Table 4:** factors influencing acceptability of the COVID-19 vaccine by hospital staff

			Univariable analysis		Multivariable analysis	
**Characteristic**	**Acceptance n (%)**	**Reluctance n (%)**	**OR(95% CI)**	**p**	**aOR(95% CI)**	**p**
**Sex**						
Male	106 (59.6)	72 (40.4)	Ref	0.783		
Female	128 (58.2)	92 (41.8)	0.94 (0.63-1.41)			
**Age groups**						
20-34	80 (61.5)	50 (38.5)		0.736		
35-49	97 (57.7)	71 (42.3)				
>=50 years	57 (57)	43 (43)				
**Years of service**						
0-9	65 (55.1)	53 (44.9)		0.551		
10-19	72 (62.1)	44 (37.9)				
>=20 years	97 (59.1)	67 (40.9)				
**Influenza vaccine**						
Accepts	137 (70.3)	58 (29.7)	Ref	0.000	Ref	
Refusal	97 (47.8)	106 (52.2)	2.58 (1.70-3.89)	0.000	2.58 (1.69-3.94)	0.000
**Academic training**						
Medical and related	105 (51.5)	99 (48.5)	Ref		Ref	
Non-medical	129 (66.5)	65 (33.5)	0.74 (0.61-089)	0.002	0.63 (0.12-3.21)	0.579
**Function**						
Physicians and Pharmacists	17 (43.6)	22 (56.4)	0.56 (0.30-1.04)	0.067	0.57 (0.10-3.09)	0.520
Paramedicals*	88 (53.3)	77 (46.7)	0.78 (0.61-0.99)	0.045	0.96 (0.20-4.66)	0.968
Cleaning staff	43 (67.2)	21 (32.8)	1.43 (0.88-2.32)	0.136		
Administrators	86 (66.2)	44 (33.8)	1.37 (1.01-1.85)	0.038	098 (0.52-1.91)	0.998

Ref: reference category; CI: confidence interval; OR: odds ratio; aOR: adjusted odds ratio; * Paramedicals: nurses, health technicians, orderlies

## Discussion

Our study showed that the discovery of a vaccine against COVID-19 feeds both hope and apprehension among general hospital staff. Vaccination, although recognized as a source of collective protection, was rejected by nearly half of the participants mainly because of its potential side effects and the doubts about the vaccine's efficacy even among medical and paramedical staff. People who refuse the influenza vaccine are the most reluctant to get the COVID-19 vaccine.

Apprehension about vaccination is not new and is not limited to health workers, as fear of the adverse effects of vaccines would have outweighed fear of the disease [10]. In the general population, these refusals would be due to misinformation on the internet or in the media, loss of trust in experts, cultural beliefs, religious or moral convictions, and pseudo-scientific beliefs [11,12].

Pending the discovery of the COVID-19 vaccine, health authorities are encouraging health workers to get mass influenza vaccination to retain staff and limit the risk of disruption to services [13]. The objective would be to reach 75% vaccination coverage among health care workers compared to the current coverage of 20% in Switzerland [14], 35% in France [15] and 41% in Quebec [16]. This reluctance to be vaccinated is long-standing and of multifactorial origin [12].

Studies among health care workers have shown that the main reasons for refusing occupational vaccination were the belief that vaccination is not useful [17-19], the fear of side effects [17-19], the belief that one is not at risk [20], being too young or healthy [20], the use of other preventive means [19] and the poor knowledge of the disease and the vaccine [19]. The main reasons for approval of vaccines by health workers were to protect patients [18-20], to protect oneself [18-20], and to protect one's family [19,20]. Most of these factors were objectified by our study as the main reasons for reluctance of the COVID-19 vaccine. We did not find any age-related difference in the vaccination decision; however, we did find a greater sense of indifference to the pandemic among the youngest, which can be explained by the belief that they are healthy and are not at risk [21].

Recent studies, mainly in the United States, have shown that nearly 70% of the general population accept COVID-19 vaccine. The percentage of those who accepted vaccination was 67-69% in the USA [22,23], 62-75% in France [24,25], 70% in Germany, 79% in the United Kingdom, 80% in Denmark, 73% in the Netherlands and 75% in Portugal [25]. Another United States of America study showed that 57.6% intended to be vaccinated, 31.6% were not sure and 10.8% refused the vaccine [26].

Acceptability factors for the COVID-19 vaccine by general population were the belief that the pandemic will last for years [27], the feeling of being at high risk of contracting the disease [22], to protect oneself, one's family and society [22,27]. Factors that contributed to the rejection of the COVID-19 vaccine were fear of new vaccines, fear of side effects [25-27], doubts about the efficacy of the vaccine [28], young age [26], low educational attainment [26], not having received the influenza vaccine in the previous year [26], not fearing contamination with COVID-19 virus [28] and the belief in conspiracies related to COVID-19 [29]. A study conducted in China assessing the acceptability of COVID-19 vaccine by nurses showed that only 40% accepted the vaccine [30]. These were those with chronic disease (OR: 1.83), managing patients with COVID-19 (OR: 1.63) and those vaccinated against influenza in 2019 (OR: 2.03). The two main reasons for refusal were doubts about the vaccine's efficacy and fear of side effects [30].

Among the factors that can influence the spread of the virus, we noted that the spiritual and the divine had its place among our participants alongside more objective and current scientific factors. Tunisia would be protected by its saints who confer psychological immunity in the collective memory of Tunisians [31]. But these divine beliefs did not influence the vaccination decision. The decision to vaccinate against COVID-19 by physicians, pharmacists and paramedics seems to be influenced by their expertise in this field (academic training), probably due to their fear of injecting themselves with a product that would be designed quickly with shortened testing phases and not conform to the standards they are familiar with.

The literature abounds with actions and recommendations to protect and improve the immunization coverage of health care workers. Concerted, transparent, clear and effective communication could restore confidence in immunization by conveying messages based on scientific knowledge [8,10,14]. Multimedia tools (clips, social networks) should be used to strengthen communication, fight against “fake news” [10,14], and better target young people [20]. These vaccination promotion campaigns should be multidimensional, highly motivational and adapted to the socio-professional categories [19]. Some authors advocate better organization of the structures responsible for vaccination to provide better information on the justification for vaccination [11,32]. Finally, other authors recommend making certain vaccinations compulsory [19,33], and setting up a system of state compensation for damage resulting from these vaccinations [34].

Our study has shown the importance of organizing vaccination awareness campaigns among hospital staff. During these campaigns, it should be emphasized that it is a means of individual and collective protection, it protects us, our families and our patients. We also recommend communicating about the development stages of COVID-19 vaccines and informing about their adverse effects as they are discovered. As a last resort, we propose to institute mandatory vaccination against COVID-19 for all hospital personnel with compensation for potential side effects.

This study was carried out at the end of the first wave (lull period) and reflects an image closely related to the social representation of the coronavirus at that time, an image that could change as the pandemic evolves. Nevertheless, our study also has several strong points since our sample was representative of hospital staff, data collection was done during an interview conducted by the same clinical psychologist and the assessment of factors that could at any time influence the decision to vaccinate.

## Conclusion

Our study showed that many hospital staff are reluctant to be vaccinated against COVID-19. Vaccination apprehension does not seem to spare the COVID-19 vaccine despite the severity of this disease, its lethality and its rapid spread. As with the influenza vaccine, fear of side effects and doubts about the efficacy of the vaccine are the main causes of reluctance. Vaccination information and awareness campaigns should be conducted among hospital staff. In addition to safety, these campaigns should emphasize the efficacy of the new vaccines that allow hospital staff to protect themselves, their families and their patients. The objective of these campaigns is to achieve high coverage with the COVID-19 vaccine to limit the spread of the disease among hospital staff and to limit the risk of service disruption.

### What is known about this topic

- Health care workers are reluctant to be vaccinated against influenza;
- The main causes of reluctance to vaccinate against influenza are the belief that vaccination is not useful and the fear of side effects;
- The main reasons for approval of vaccines by health workers were to protect patients, to protect oneself and to protect one's family.

### What this study adds

- Health care workers are reluctant to be vaccinated against COVID-19;
- Those who have already been vaccinated against influenza are those who are most willing to be vaccinated against COVID-19;
- Those who refuse the influenza vaccine are the most reluctant to be vaccinated against COVID-19. The main causes of reluctance to vaccinate against COVID-19 are the belief that vaccination is not useful and the fear of side effects.
